# Arl3 and RP2 regulate the trafficking of ciliary tip kinesins

**DOI:** 10.1093/hmg/ddx143

**Published:** 2017-04-21

**Authors:** Nele Schwarz, Amelia Lane, Katarina Jovanovic, David A. Parfitt, Monica Aguila, Clare L. Thompson, Lyndon da Cruz, Peter J. Coffey, J. Paul Chapple, Alison J. Hardcastle, Michael E. Cheetham

**Affiliations:** 1 UCL Institute of Ophthalmology, London EC1V 9EL, UK; 2 Institute of Bioengineering, School of Engineering and Materials Science, Queen Mary University of London, London E1 4NS, UK,; 3 Moorfields Eye Hospital, London EC1V 2PD, UK; 4 William Harvey Research Institute, Barts and the London School of Medicine, Queen Mary University of London, London EC1M 6BQ, UK

## Abstract

Ciliary trafficking defects are the underlying cause of many ciliopathies, including Retinitis Pigmentosa (RP). Anterograde intraflagellar transport (IFT) is mediated by kinesin motor proteins; however, the function of the homodimeric Kif17 motor in cilia is poorly understood, whereas Kif7 is known to play an important role in stabilizing cilia tips. Here we identified the ciliary tip kinesins Kif7 and Kif17 as novel interaction partners of the small GTPase Arl3 and its regulatory GTPase activating protein (GAP) Retinitis Pigmentosa 2 (RP2). We show that Arl3 and RP2 mediate the localization of GFP-Kif17 to the cilia tip and competitive binding of RP2 and Arl3 with Kif17 complexes. RP2 and Arl3 also interact with another ciliary tip kinesin, Kif7, which is a conserved regulator of Hedgehog (Hh) signaling. siRNA-mediated loss of RP2 or Arl3 reduced the level of Kif7 at the cilia tip. This was further validated by reduced levels of Kif7 at cilia tips detected in fibroblasts and induced pluripotent stem cell (iPSC) 3D optic cups derived from a patient carrying an *RP2* nonsense mutation c.519C > T (p.R120X), which lack detectable RP2 protein. Translational read-through inducing drugs (TRIDs), such as PTC124, were able to restore Kif7 levels at the ciliary tip of RP2 null cells. Collectively, our findings suggest that RP2 and Arl3 regulate the trafficking of specific kinesins to cilia tips and provide additional evidence that TRIDs could be clinically beneficial for patients with this retinal degeneration.

## Introduction

Primary cilia are small, hair-like organelles on the surface of most cells. They act as environmental sensors and organize signalling hubs, such as PDGF, Hedgehog, Wnt and Hippo cascades. In the retina, the photoreceptor outer segments are highly specialized cilia that detect light and initiate the phototransduction signalling cascade. Cilia assembly and maintenance requires coordinated intraflagellar transport (IFT). IFT is mediated by kinesin and dynein driven bi-directional movement of cargo proteins along the ciliary axoneme and defects in cilia assembly and/or function are the underlying cause of many genetic disorders, classified as ciliopathies (1–5).

In cilia the two main anterograde motors are heterotrimeric Kif3a, which is complexed with either Kif3b or Kif3c and Kinesin-associated polypeptide 3 (KAP3), and homodimeric Kif17. Systemic deletion of Kif3a and Kif3b are embryonic lethal in mice (6–8), whereas Kif3c knockout mice display no discernable phenotype, suggesting a level of redundancy among Kif3 subunits (9). However, targeted retina-specific knockdown of Kif3a results in mislocalisation of opsin and photoreceptor cell death (10,11) and expression of a dominant negative form of the Kif3b subunit during early development in *Xenopus* rod photoreceptors causes disrupted photoreceptor organization and leads to cell death (12), implying a vital function for Kif3a and Kif3b in photoreceptor development and maintenance.

In contrast, the role of Kif17 in vertebrate ciliogenesis is not clear. Kif17 is the mammalian homologue of the *C. elegans* molecular motor protein osmotic avoidance abnormal protein 3 (OSM3). In *C. elegans* both types of kinesin-2 motors, Kif3 and OSM3 (Kif17), cooperate to build the axoneme core of sensory cilia on chemosensory neurons, but only OSM3 drives the elongation of the distal segments (13,14). In zebrafish photoreceptor cells Kif17 interacts with Kif3a/b and KAP and localizes to the connecting cilium and the periciliary ridge, where it potentially mediates transport of IFT proteins in photoreceptors (15); however, several zebrafish Kif17 models have been established that develop different phenotypes. Morpholino-mediated Kif17 knockdown, or expression of dominant-negative Kif17 constructs resulted in failure of photoreceptor outer segment development, but had no impact on embryogenesis or other ciliated organs (15,16). Conversely, a chemically induced Kif17 knockout in zebrafish resulted only in shortened olfactory cilia with no effect on opsin localisation in photoreceptors, suggesting Kif17 plays a minor role, if any, in ciliogenesis and retinal development (17,18). Furthermore, Kif17 knockout mice are viable with no significant anatomical defects, but displayed decreased levels of N-methyl-D-aspartate (NMDA) receptor subunits 2A and B (19). Rod-specific knockout of both Kif3/Kif17 demonstrated that the two motors do not appear to compensate for each other, either in building photoreceptor outer segments or in transporting photoreceptor specific cargo proteins (20). Kif17 accumulates at the tips of cilia *in vivo* and *in vitro*, but the significance of this is not clear (21,22). In contrast, the cilia tip compartment is shaped by the kinesin Kif7, which regulates cilia length and stability; however, it is currently unclear how Kif7 localizes to cilia, since Kif7 does not display any motor function on microtubules (23).

In *C. elegans*, a model was proposed in which OSM3 (Kif17) co-operates with the small GTPases Arl13b and Arl3 in IFT-mediated cilia formation. It was proposed that Arl13b stabilizes the binding between the IFT-A and IFT-B complex, while Arl3 regulates the association between the IFT-B complex and OSM3 (24), suggesting GTPase mediated regulation of the IFT machinery and Kif17. Arl3 is regulated by its guanine nucleotide exchange factor (GEF) Arl13b (25) and GTPase activating protein (GAP) Retinitis Pigmentosa 2 (RP2) (26). In humans, mutations in *ARL13b* cause Joubert Syndrome (27), whereas mutations in *RP2* cause X-linked RP (26,28,29). In addition, loss of Arl3 causes cilia defects in multiple tissues in mice (30). Dysregulation of Arl3, through siRNA-mediated knockdown of RP2 or expression of a constitutively active mimic of Arl3, resulted in dispersal of vesicle cycling cargo from the Golgi complex to the cilium, including the intraflagellar transport protein IFT20 (31). In addition, a pool of RP2 and Arl3 co-localize to the ciliary apparatus, namely the basal body and the associated centriole at the base of the photoreceptor cilium (31–34), implying a role for RP2 and Arl3 in primary cilia protein trafficking, possibly via the regulation of kinesin motor proteins.

We therefore investigated the hypothesis that Arl3 and its GAP, RP2, co-operate with kinesin motor proteins to regulate the cilia protein trafficking. Here, we show that loss of Arl3 or RP2 cause a reduction of Kif17 and Kif7 levels at the ciliary tip. Furthermore, Arl3 and RP2 were able to complex with Kif17 and Kif7, suggesting a function for Arl3 and RP2 in regulating ciliary tip kinesin localisation. The observed loss of Kif7 at cilia tips was rescued in fibroblasts and induced pluripotent stem cell (iPSC)-derived 3D optic cup photoreceptors obtained from a patient carrying an RP2 non-sense mutation with the translational read-through inducing drug (TRID), PTC124.

## Results

### RP2 and Arl3 are required for the localisation of GFP-Kif17 to the cilia tip

**Figure 1:**
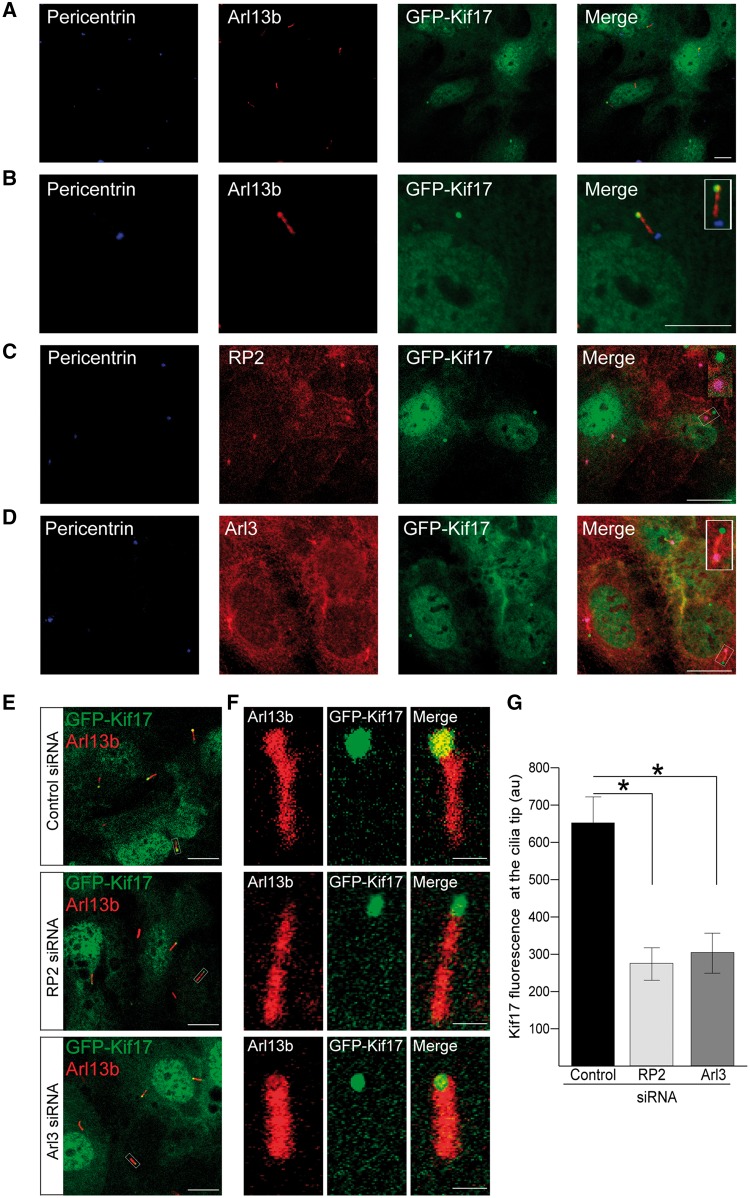
RP2 and Arl3 mediate GFP-Kif17 cilia tip localisation. (**A**) In stable GFP-Kif17 hTERT-RPE cells Kif17 (green) localises to the nucleus, the cytoplasm and to cilia tips, as indicated by the cilia marker Arl13b (red) and the basal body marker pericentrin (blue). Scale bar 10 μm. (**B**) Zoomed image of GFP-Kif17 hTERT-RPE cell line. Inset shows a zoomed in image of the cilia tip. Scale bar 10 μm. (**C**) RP2 (red) localises to the basal body (pericentrin, blue) of cilia in GFP-Kif17 hTERT-RPE cells. Scale bar 10 μm. (**D**) Arl3 (red) localises to the basal body (pericentrin, blue) and the along the ciliary axoneme in GFP-Kif17 hTERT-RPE cells. Scale bar 10 μm. (**E**) siRNA-mediated knockdown of RP2 or Arl3 decreases GFP-Kif17 at cilia tips. Cilia marker Arl13b (red). Scale bar 10 μm. (**F**) Zoomed image of GFP-Kif17 at cilia tips following control, RP2 or Arl3 siRNA transfection. Cilia marker Arl13b (red). Scale bar 1 μm. (**G**) Quantification of GFP-Kif17 fluorescence at cilia tips after control, RP2 or Arl3 siRNA transfection. n = 3 independent experiments. *P ≤ 0.05, values are mean ± SEM.

Arl3, together with its GEF Arl13b, have been implicated in the assembly and maintenance of the IFT motor complex, kinesin-2 and OSM3 traffic in *C. elegans* (24). We therefore investigated whether Arl3 and its regulatory protein RP2, play a role in mammalian Kif17 localisation. We created an hTERT-RPE cell line that stably expresses full-length human GFP-Kif17. In these stable cells GFP-Kif17 localized to the nucleus, cytoplasm and the ciliary tip, as described previously (21,22) (Fig. 1A and B). RP2 and Arl3 localized to the basal body (Fig. 1C and D). In addition, Arl3 staining was observed along the ciliary axoneme and co-localized with GFP-Kif17 at cilia tips (Fig. 1D). This localisation of RP2 at the basal body is consistent with earlier reports for the endogenous protein (31,33,34); however, RP2-GFP can enter cilia (33,35). To investigate RP2 localisation further, we used structured illumination super resolution microscopy (SIM) to study the fine detail of endogenous RP2 localisation in hTERT-RPE cells. This revealed that, in addition to accumulating at the basal body, a pool of endogenous RP2 was present along the ciliary axoneme with occasional more intense punctate staining and some staining at the cilia tip (Supplementary Material, Fig. S1).

siRNA-mediated RP2 and Arl3 depletion (31,36) resulted in a robust target protein knockdown in the GFP-Kif17 cell line, without affecting the overall expression levels of GFP-Kif17 (Supplementary Material, Fig. S2A). Knockdown of either RP2 or Arl3 significantly reduced the level of GFP-Kif17 at the cilia tips in GFP-Kif17 stable hTERT-RPE cells, without affecting the nuclear or cytoplasmic localisation of GFP-Kif17 in these cells, compared to control siRNA (Fig. 1E and F). Therefore, the role of Arl3 on Kif17 cilia traffic appears to be conserved. Loss of Arl13b causes severe cilia shortening and structural defects of the ciliary axoneme (37); therefore, it was not possible to determine the effect of Arl13b knockdown on GFP-Kif17 cilia tip localisation in this model.

### Arl3 and RP2 mediate Kif7 localisation to cilia tips

**Figure 2:**
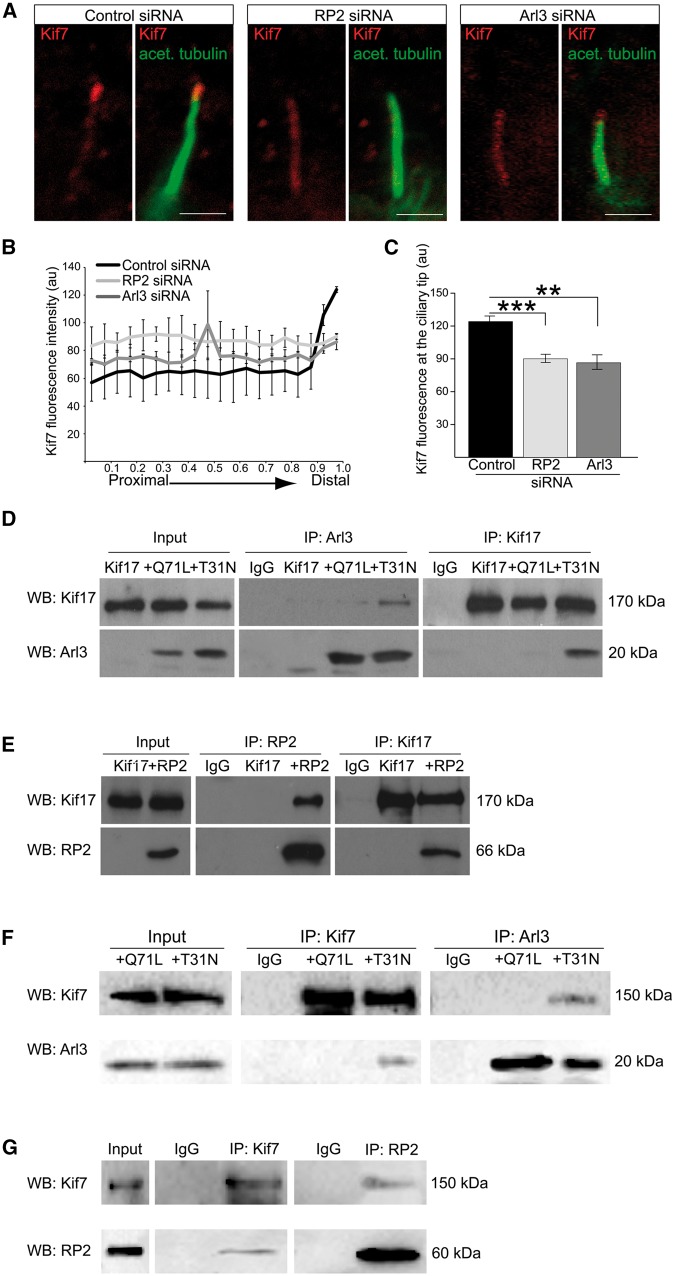
RP2 and Arl3 mediate the localisation of Kif7 to cilia tips. (**A**) siRNA-mediated knockdown of RP2 or Arl3 in hTERT-RPE cells decreases Kif7 (red) at cilia tips. Cilia marker acetylated α-tubulin (green). Scale bar 1 μm. (**B**) Fluorescence intensity of Kif7 along the axoneme, normalised for cilium length. n = 3 independent experiments, with a total of 90 cilia measured for each siRNA treatment. For RP2 siRNA, P ≤ 0.0001 for the last measurement of the cilia tip. For Arl3 siRNA, P ≤ 0.05 and P ≤ 0.001, for the distal part of cilia. Values are mean ± SEM. (**C**) Quantification of Kif7 fluorescence at cilia tips. n = 3 independent experiments, with a total of 90 cilia measured for each siRNA treatment. ***P ≤ 0.0001, **P ≤ 0.001, values are mean ± SEM. **(D-G)** Reciprocal co-IPs of different combinations; (D) GFP-Kif17 with the myc-tagged Arl3 conformational mimics Arl3-GTP (Q71L) and Arl3-GDP (T31N) shows that GFP-Kif17 preferentially binds to Arl3-T31N. (E) GFP-Kif17 binds to RP2. (F) V5-Kif7 with the myc-tagged Arl3 conformational mimics Arl3-GTP (Q71L) and Arl3-GDP (T31N) shows that Kif7 preferentially binds to Arl3-T31N. Kif7 and Arl3 were immunopurified with the V5 and myc antibody, respectively. (G) V5-Kif7 binds to RP2. Kif7 and RP2 were immunopurified with the V5 antibody and GFP-trap magnetic beads, respectively.

Since Arl3 and RP2 are important for the ciliary tip localisation of Kif17, we investigated whether Arl3 and RP2 influence the localisation of other kinesins that are present in the ciliary tip compartment. Kif7, a kinesin family-4 member, regulates the dynamics of microtubule plus ends and thereby controls the structure and organisation of primary cilia tips (23). To determine whether RP2 and Arl3 mediate the localisation of Kif7 to the ciliary tip, we used immunocytochemistry (ICC) to analyse Kif7 localisation in hTERT-RPE cells treated with control, RP2 or Arl3 siRNA. In control hTERT-RPE cells endogenous Kif7 localized to the tips of cilia, as reported previously (23); however, siRNA-mediated knockdown of RP2 and Arl3 caused a significant reduction of Kif7 at the cilia tip (Fig. 2A–C).

### Arl3 and RP2 interact with Kif17 and Kif7, but not other kinesins

To determine whether Arl3 and Kif17 could interact in mammalian cells, full-length human myc-Kif17 was co-transfected with the constitutively active Arl3-GTP conformational mimic Arl3-Q71L-vsv, or the Arl3-GDP mimic, Arl3-T31N-vsv. Reciprocal co-immunopurification (co-IP) experiments showed that Kif17 co-purified preferentially with the Arl3 GDP-form, Arl3-T31N (Fig. 2D). To investigate whether RP2 can also bind Kif17, cells were transfected with RP2-GFP and myc-Kif17 and reciprocal co-IPs were performed, these showed that RP2 and Kif17 also co-purify (Fig. 2E). The potential of Kif17 to form a complex with both Arl3-GDP and RP2 was investigated and the reciprocal co-IPs showed that RP2 competed with Arl3-GDP for Kif17 binding. This shows that the three proteins do not form a heterocomplex in this assay (Supplementary Material, Fig. S3). These data suggest that regulation of Kif17 cilia trafficking could be mediated by a direct interaction with RP2 and Arl3 complexes.

To test the hypothesis that RP2 and Arl3 play a general role in ciliary kinesin protein trafficking, reciprocal co-IPs were performed from lysates of cells transfected with V5-Kif7 with either Arl3-Q71L-myc, Arl3-T13N-myc or RP2-GFP. These co-IPs revealed that, similar to Kif17, Kif7 specifically co-purified with the GDP conformational mimic of Arl3 (Ar3-T31N), but not the GTP-bound conformation (Arl3-Q71L), similar to Kif17 (Fig. 2F). In addition, Kif7 also co-purified with RP2 (Fig. 2G). Kif17 is a plus end-directed, homodimeric kinesin and together with Kif3a/b belongs to the kinesin-2 family (38). We therefore investigated whether Arl3 and RP2 affect the ciliary trafficking of Kif3a by co-transfecting cells with GFP-tagged Kif3a and myc-Arl3 (Q71L and T31N) or untagged RP2; however, neither RP2 nor Arl3 co-purified with Kif3a (Supplementary Material, Fig. S4A and B). The lack of binding of RP2 and Arl3 to Kif3a does not appear to be due to the fusion protein as GFP-Kif3a successfully immunopurified Kif3b, which together with Kif3a and KAP forms the kinesin-2 trimer (Supplementary Material, Fig. S4C). These findings suggest that RP2 and Arl3 function specifically in the import, or export, of distinct ciliary tip kinesins.

### Kif7 functions up-stream of Kif17

**Figure 3:**
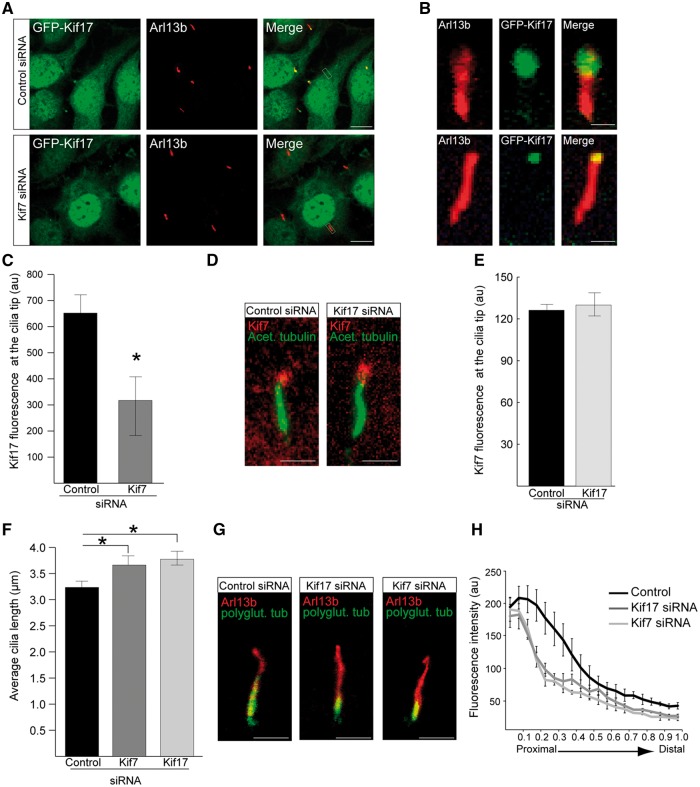
Kif7 functions upstream of Kif17 in cilia and both kinesins contribute to cilia stability. (**A**) siRNA-mediated knockdown of Kif7 in GFP-Kif17 hTERT-RPE cells reduces GFP-Kif17 at cilia tips. Scale bar 10 μm. (**B**) Zoomed in image of GFP-Kif17 cilia localisation following control or Kif7 siRNA treatment. Scale bar 1 μm. (**C**) Quantification of GFP-Kif17 fluorescence at the cilia tip following siRNA treatment. n = 3 independent experiments with 150 cilia measured for each siRNA. *P ≤ 0.05, values are mean ± SEM. (**D**) hTERT-RPE cells stained for Kif7 (red) and acetylated α-tubulin (green) following siRNA treatment. Scale bar 1 μm. (**E**) Quantification of Kif7 fluorescence at the cilia tip following siRNA treatment. n = 3 independent experiments with 150 cilia analysed for each siRNA. (**F**) Cilia length in hTERT-RPE cells treated with Kif7 and Kif17 siRNA is increased compared to controls. n = 3 independent experiments with 150 cilia measured for each siRNA. *P ≤ 0.05, values are mean ± SEM. (**G**) hTERT-RPE cilia treated with Kif17 or Kif7 siRNA show decreased levels of polyglutamylated tubulin (green) compared to controls. Cilia marker, Arl13b (red). Scale bar 1 μm. (**H**) Fluorescence intensity of polyglutamylated tubulin, normalised for cilium length. n = 3 independent experiments, with a total of 90 cilia measured for each siRNA treatment. For Kif7 and Kif17 siRNA, P ≤ 0.05 for the proximal part of cilia. Values are mean ± SEM.

It is unknown if Kif17 and Kif7 are part of the same or parallel pathways in cilia function. To address this question, siRNAs against Kif7 and Kif17 were used to determine their hierarchy. siRNA-mediated knockdown of Kif7 in GFP-Kif17 hTERT-RPE cells significantly reduced levels of GFP-Kif17 at the cilia tip (Fig. 3A–C); however, knockdown of Kif17 in hTERT-RPE cells had no effect on Kif7 ciliary tip localisation, suggesting that Kif7 is important for the localisation of Kif17, but not vice versa (Fig. 3D and E). The effect of reduced levels of Kif7 and Kif17 on cilia morphology was analysed following treatment of hTERT-RPE cells with siRNAs against Arl3, RP2, Kif17 and Kif7. siRNA against Kif17 resulted in robust target protein knockdown and significantly reduced GFP-Kif17 fluorescence at cilia tips (Supplementary Material, Fig. S2B and C). Cilia were present on approximately 65% of cells, irrespective of siRNA (Supplementary Material, Fig. S2D and E), but cilia were significantly longer only in Kif7 and Kif17 knockdown cells compared to control siRNA treated cells (Fig. 3F). This is in agreement with observations of increased cilia length in *Kif7^-/-^* mouse embryonic fibroblasts (23).

The microtubules of the cilia axoneme undergo several post-translational modifications, such as acetylation and polyglutamylation, which confer microtubule stability (39). Kif17 localizes to microtubule plus ends where it promotes cytoplasmic microtubule acetylation (40). In addition, loss of Kif7 has previously been shown to affect the level of tubulin acetylation and polyglutamylation in cilia, suggesting that Kif7 promotes the stability of the ciliary axoneme (23). Since Kif7 and Kif17 both localize to cilia tips and loss of both proteins alters cilia length, we examined whether loss of Kif17 also affects ciliary microtubule stability.

Line-scan analysis of the fluorescent intensity of polyglutamylated tubulin following knockdown of Kif17 and Kif7 showed a significant reduction in polyglutamylation levels from the ciliary base to the distal tip of cilia compared to controls (Fig. 3G and H), suggesting that Kif17 stabilizes cilia independently of Kif7. Knock-down of Kif7 or Kif17 had no effect on total cellular polyglutamylation levels (Supplementary Material, Fig. S5A and B), suggesting that Kif7 and Kif17 specifically stabilize cilia microtubules.

To determine whether loss of RP2 or Arl3 also effect cilia stability, line-scan analysis of the fluorescent intensity of polyglutamylated tubulin was performed in hTERT-RPE cells treated with siRNA against RP2 or Arl3 and compared to controls. In addition, we analysed fibroblasts from an individual with X-linked RP with an *RP2* nonsense mutation R120X (c.519C > T), which abolishes RP2 protein expression (41). RP2 and Arl3 siRNA-mediated knockdown cells, as well as RP2 patient fibroblasts showed the same levels of polyglutamylated tubulin throughout the ciliary axoneme as controls (Supplementary Material, Fig. S5C–F). This indicates that the indirect reduction of Kif7 and Kif17 at cilia tips caused by absence of RP2 or Arl3 is not sufficient to result in detectable cilia instability. In contrast, targeted knockdown of Kif7 or Kif17 does affect cilia stability.

These findings suggest that both Kif7 and Kif17 are required for cilia stability, and that Kif7 regulates Kif17 localisation to the cilia tip, either directly or by organising the ciliary tip compartment in a way that enables Kif17 to accumulate at the tip.

### The effect of RP2 and Arl3 on ciliary tip kinesins is independent of IFT-B2 complexes

A recent study has shown that the cilia entry of Kif17 is dependent on its nuclear localisation sequence and the interaction with the IFT-B complex (42). Therefore, we examined if Arl3 and RP2 could be mediating the reduction of Kif17 in cilia through IFT-B. ICC staining of the IFT-B2 component IFT57 in control and RP2 R120X patient fibroblasts and revealed that loss of RP2 had no effect on cilia localisation of IFT57, suggesting that RP2 and Arl3 mediate Kif17 cilia localisation independently of IFT57 (Supplementary Material, Fig. S6).

### RP2 and Arl3 are required for the localisation of the sonic hedgehog (Shh) protein Gli3

**Figure 4:**
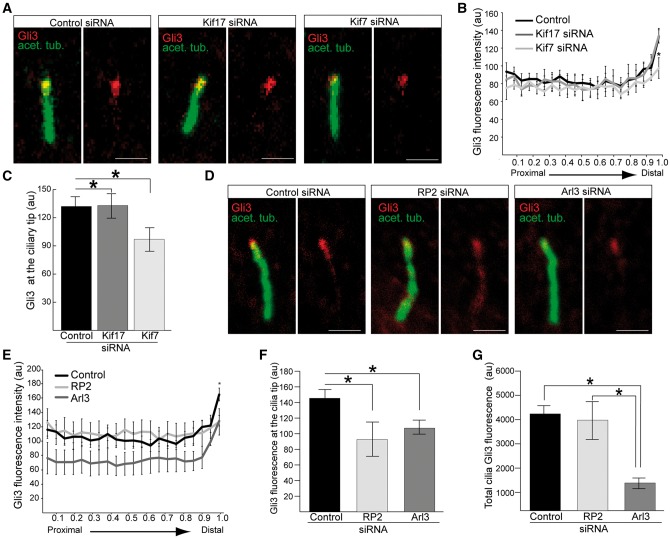
Loss of RP2 and Arl3 mediated reduction of Kif7 affects the ciliary tip localisation of Gli3. (**A**) hTERT-RPE cells treated with siRNA against Kif7, but not Kif17, reduces Gli3 (red) at cilia tips. Cilia marker acetylated α-tubulin (green). Scale bar 1 μm. (**B**) Fluorescence intensity of Gli3, normalised for cilium length. n = 3 independent experiments, with a total of 90 cilia measured for each siRNA treatment. *P ≤ 0.05, values are mean ± SEM. (**C**) Quantification of Gli3 fluorescence at the cilia tip following Kif7 or Kif17 siRNA treatment. n = 3 independent experiments with 150 cilia analysed for each siRNA. *P ≤ 0.05, values are mean ± SEM. (**D**) hTERT-RPE cells treated with siRNA against RP2 or Arl3 reduces Gli3 (red) at cilia tips. Cilia marker acetylated α-tubulin (green). Scale bar 1 μm. (**E**) Fluorescence intensity of Gli3, normalised for cilium length. n = 3 independent experiments, with a total of 90 cilia measured for each siRNA treatment. For RP2 and Arl3 siRNA, *P ≤ 0.05 for the distal part of cilia. For Arl3 siRNA, P ≤ 0.05 for the proximal part of cilia. Values are mean ± SEM. (**F**) Gli3 fluorescence is reduced at the cilia tip following RP2 or Arl3 siRNA treatment. n = 3 independent experiments with 150 cilia analysed for each siRNA. *P ≤ 0.05, values are mean ± SEM. (**G**) Total Gli3 fluorescence is reduced in cilia in Arl3 knockdown cells, compared to RP2 knockouts and control. n = 3 independent experiments with 150 cilia analysed for each siRNA. *P ≤ 0.05, values are mean ± SEM.

Previous studies have identified Kif7 as a critical regulator of Hedgehog cascades, where Kif7 functions to shape the cilia compartment to enable the enrichment of Gli proteins (23,43–45). Therefore, we investigated whether loss of Kif7 or Kif17 affected the localisation of the Sonic hedgehog (Shh) protein Gli3. In hTERT-RPE cells treated with Kif7 siRNA Gli3 levels at the tip of the cilium were decreased compared to cells treated with control or Kif17 siRNA (Fig. 4A–C), suggesting that Kif17 is not required for Shh signalling.

A high-content cilia screen showed that siRNA-mediated loss of Arl3 leads to a reduction in ciliary Shh signalling, caused by impaired Gli3 transport (46). We therefore reasoned that since RP2 and Arl3 play a role in ciliary tip kinesin traffic they could also influence Gli3 protein localisation. Gli3 staining was reduced at the ciliary tip in Arl3 and RP2 depleted cells compared to controls, with Gli3 re-distributed along the ciliary axoneme of RP2 siRNA treated cells (Fig. 4D–F). In Arl3 knockdown cells the overall level of Gli3 in cilia was reduced compared to RP2 and control knockdown cells (Fig. 4G), implying that Arl3 plays an important role in Gli3 cilia function.

### Translational read-through inducing drugs are able to restore the ciliary localisation of Kif7 and Gli3

**Figure 5:**
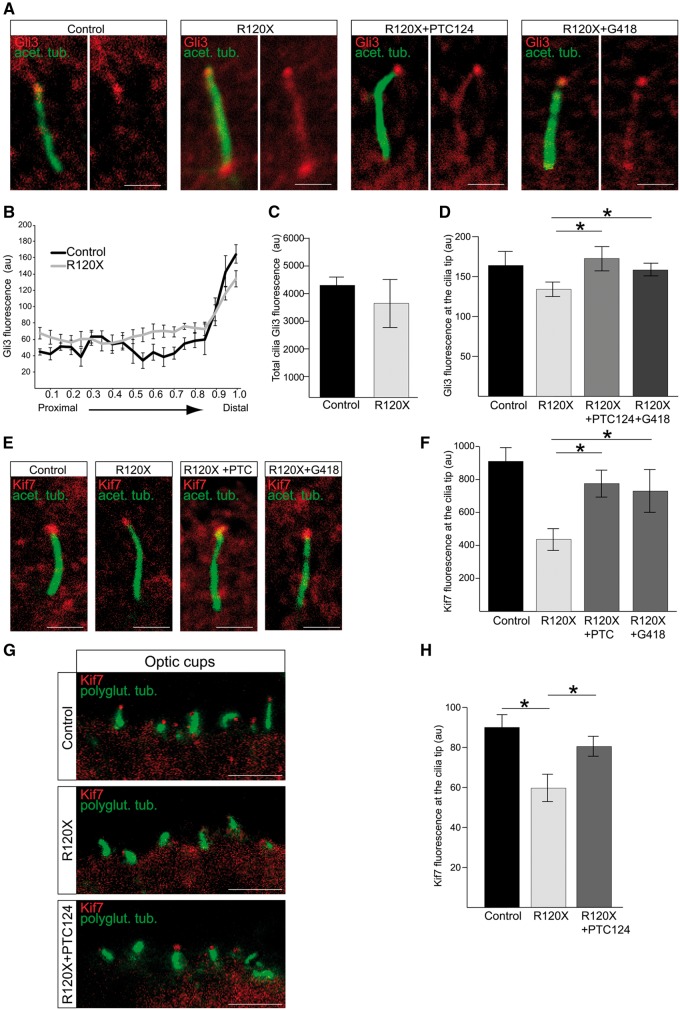
Treatment with TRIDs rescues Kif7 cilia localisation in RP2 R120X fibroblasts and 3D optic cups. (**A**) Ciliary tip localisation of Gli3 is restored in RP2 R120X fibroblasts. Cells were treated with a single 24-h dose of either 750 μM G418 or 10 μg/ml PTC124 and stained for Gli3 (red) and acetylated α-tubulin (green). Scale bar 1 μm. (**B**) Fluorescence intensity of Gli3, normalised for cilium length in control and RP2 R120X fibroblasts shows an increase of Gli3 fluorescence along the ciliary axoneme and reduction of Gli3 at the cilia tips. For RP2 R120X, P ≤ 0.05 at the proximal, central and distal part of cilia. n = 3 independent experiments, with a total of 90 cilia measured. Values are mean ± SEM (**C**) Total cilia Gli3 fluorescence levels in cilia from RP2 R120X fibroblasts are comparable to control cilia. (**D**) Quantification of Gli3 fluorescence at cilia tips. n = 3 independent experiments, with a total of 90 cilia measured. *P ≤ 0.05, values are mean ± SEM. (**E**) Treatment of RP2 R120X fibroblasts with a single 24-h dose of either 750 μM G418 or 10 μg/ml PTC124 restores Kif7 (red) levels at cilia tips. Cilia marker acetylated α-tubulin (green). Scale bar 1 μm. (**F**) Quantification of Kif7 fluorescence intensity at cilia tips in untreated R120X fibroblasts compared to control cells. This RP2-null cellular phenotype was reversed with treatment of either G418 or PTC124. n = 3 independent experiments, with a total of 90 cilia measured. *P ≤ 0.05, values are mean ± SEM. **(G)** PTC124 treated and untreated RP2 R120X iPSC-derived 3D optic cups were stained for Kif7 (red) and polyglutamylated tubulin (green) and compared to control 3D optic cups. Scale bar 5 μm. (**H**) Kif7 levels are restored at cilia tips in 3D optic cups following treatment with three doses of 10 μg/ml PTC124 over the course of seven days. n ≥ 60 cilia analysed. *P ≤ 0.05, values are mean ± SEM.

The importance of RP2 for protein trafficking to the cilia tip was validated in an RP2 null fibroblast cell model, derived from an individual with the R120X RP2 mutation (41). Gli3 localisation at cilia tips was significantly reduced in R120X fibroblasts and accumulated at the basal body and along the length of the ciliary axoneme, compared to control fibroblasts (Fig. 5A and B); however, the loss of RP2 did not affect the overall level of Gli3 in cilia (Fig. 5C). These findings are in agreement with the findings in the hTERT-RPE RP2 knockdown cells and suggest that RP2 is not required for Gli3 entry into or exit from cilia.

The c.519C > T *RP2* mutation results in a premature termination codon (p.R120X) and we have previously shown that translational read-through inducing drugs (TRIDs), such as G418 and PTC124, can restore full length, functional RP2 protein in R120X patient fibroblasts and iPSC-derived RPE cells (41). We therefore reasoned that TRIDs could potentially reverse the observed Gli3 mislocalisation by restoring RP2 protein expression. A single 24-h dose of either G418 (750 µM) or PTC124 (10 µg/ml) significantly increased Gli3 staining at the cilia tips whilst reducing Gli3 levels along the cilia axoneme in RP2 R120X fibroblast cells (Fig. 5A and D). In addition to Gli3, cilia tip levels of Kif7 were also significantly reduced in R120X fibroblasts compared to controls (Fig. 5E and F). Treatment with both TRIDs also restored Kif7 to cilia tips, suggesting that restored Gli3 levels are downstream of Kif7, and that TRIDs can rescue this RP2-null cellular phenotype in patient fibroblasts (Fig. 5E and F).

The development of human iPSC technology has greatly enhanced the potential for understanding disease mechanisms through the directed differentiation of specific cell types affected by a particular disease directly from patient cells. To investigate retinal disease aetiology, we differentiated control and RP2 R120X patient-derived iPSC into 3D optic cups, which contain photoreceptor cells, using established protocols (47,48). RP2 R120X iPSC-derived 3D optic cups develop normally compared to control (Supplementary Material, Fig. S7A); however, RP2 R120X photoreceptors in the optic cups had reduced Kif7 staining at their cilia tips compared with controls (Fig. 5G and H). Treatment with PTC124 (10 µg/ml) over the course of one week restored Kif7 localisation at cilia tips to the level observed in control optic cup photoreceptors (Fig. 5G and H). These findings provide proof-of-concept that TRIDs are not only effective on RPE cells (41), but also in developing photoreceptors, indicating that translational read-through therapy might be clinically beneficial for RP2 stop mutations. We were not able to test the efficiency of G418 on optic cups, as this compound was toxic to the photoreceptors in the iPSC-optic cups and caused cell death (Supplementary Material, Fig. S7B).

## Discussion

Here, we have identified a novel role for Arl3 and RP2 in the trafficking of ciliary tip kinesins. Arl3 plays an important role in the traffic of lipidated proteins to cilia through its effectors Unc119 and PrBP (49–52). The involvement of Arl3 in kinesin regulation was previously suggested by genetic studies in *C. elegans* that revealed a role in OSM3 function (24). We demonstrate that the effect on Kif17 is conserved in mammals, and also involves Kif7 and RP2. The localisation of GFP-Kif17 to the ciliary tip is in accordance with previously reported *in vitro* (22) and *in vivo* models (20,21). Previous studies have shown that Kif17 contains a ciliary localisation signal (CLS) and is trafficked into primary cilia by importin beta and Ran (22,35). More recently, it was shown that the cilia entry of Kif17 was dependent on its nuclear localisation sequence (NLS) and binding to the IFT-B complex (42); however, our data suggest that the effect of Arl3 and RP2 knockdown on Kif17 and Kif7 is independent of IFT-B2. Binding of the small GTPase Rab23 to the Kif17-importin beta complex is required for Kif17 cilia localisation (53), suggesting that regulatory proteins, such as GTPases are essential for the ciliary import of Kif17. Previous studies have also suggested that Kif17 accumulates at microtubule plus ends as a result of net transport (40). Our data suggest that the ciliary trafficking proteins RP2 and Arl3 also regulate this process.

Kif7 is a kinesin-4 family protein, which regulates mammalian Hedgehog signalling by organising the ciliary tip compartment. Kif7 accumulates at microtubule plus ends, such as cilia tips and inhibits their growth (23,54). Kif17 is a kinesin motor protein whose function in cilia is not clear. In the cytoplasm, Kif17 localizes to microtubule plus ends where it promotes microtubule acetylation (40). Our study suggests that Kif17 also confers stability to the cilia axoneme by influencing levels of tubulin polyglutamylation. We show that the accumulation of Kif17 at cilia tips is dependent on the correct localisation of Kif7. This suggests that Kif7 functions upstream of Kif17, either by directly influencing Kif17 transport or by shaping the cilia tip compartment in a way that enables Kif17 accumulation at cilia tips. Lack of Kif7 at cilia tips has previously been shown to lead to dispersal of Shh proteins, such as Gli2 from cilia tips (23). We show that knockdown of Kif7, but not Kif17, reduced levels of another Shh protein Gli3 from cilia tips. This suggests that only Kif7, but not Kif17, is required to shape the cilia tip compartment, which allows the correct localisation of Shh signalling proteins. A high-content cilia screen showed that siRNA-mediated loss of Arl3 leads to a reduction in ciliary Shh signalling, caused by impaired Gli3 transport (46). In our study, siRNA-mediated knockdown of RP2 and Arl3 both reduced levels of Gli3 at cilia tips, and this is potentially a consequence of the RP2- and Arl3-dependent reduction of Kif7 at the cilia tips. Interestingly, knockdown of Arl3 effector proteins, such as Unc119 and PDE6D did not affect Kif17, Kif7 or Gli3 levels at the cilia tips (data not shown). This suggests that the effects on Arl3 and RP2 on ciliary tip kinesins and Gli proteins are independent of lipidated protein trafficking.

Arl3 appears to be essential for cilia function as deletion leads to widespread ciliopathy and high levels of embryonic lethality (30,55,56). Arl3 function is regulated through its GAP RP2 and GEF Arl13b. Interestingly, dysregulation of Arl3 through mutations in RP2 or Arl13b gives rise to different disease phenotypes. Failure to activate Arl3 function through loss of the Arl3 GEF Arl13b causes Joubert syndrome, usually with multiple organ involvement (27). On the other hand, failure to inactivate Arl3 through loss of the Arl3 GAP RP2 causes X-linked RP, without any other organ involvement (26,28,29). These different phenotypes suggest that loss of Arl3 activity leads to a broader range of phenotypes than over-activation of Arl3. Arl13b is a cilia-specific protein, and it has been proposed that active, GTP-bound Arl3 exclusively localizes within cilia were it triggers cargo release from Arl3 effectors, while GDP-bound Arl3 localizes to basal bodies or is cytoplasmic (25). Therefore, in the absence of Arl13b a pool of GDP-Arl3 could accumulate at the cilia base, while in the absence of RP2, Arl3-GTP might be enriched inside cilia. However, the mechanisms of Arl3 cilia entry and exit cilia are currently unclear. The absence of Arl13b could also lead to the accumulation of GDP-bound Arl3 inside cilia leading to the failure of Arl3-mediated cargo release from Arl3 effectors. In addition to the basal body, RP2 also localizes along the length of the ciliary compartment (Supplementary Material, Fig. S1) (33), but how RP2 ciliary entry is regulated is unclear (35,57). Loss of RP2, and failure to inactivate Arl3 function could therefore cause accumulation of Arl3-GTP inside cilia, in the cytoplasm or at the cilia base, and lead to cargo release outside of the cilia compartment, or in the wrong ciliary domain.

Kif7 and Kif17 co-purified preferentially with the GDP-conformational mimic of Arl3, suggesting that Arl3 could be involved in delivery of Kif7 and Kif17 to the ciliary base. In the absence of RP2 an increase of Arl3-GTP at the basal body and in the cytoplasm could lead to a decrease in binding efficiency of Arl3-GDP to these kinesin proteins. Based on our findings, we propose that GDP-bound Arl3 could be involved in regulating the delivery of ciliary tip kinesins to the basal body, where RP2 competes for Kif7 and Kif17 binding and releases the kinesins from GDP-Arl3, subsequently releasing Kif7 and Kif17 into the ciliary compartment (Supplementary material, Fig. S8). An alternative model is that RP2 could simultaneously stimulate the exchange of GTP for GDP on Arl3, stimulating the release of the kinesin and binding of RP2 to the released kinesin. Thereby, RP2 could function as a docking station for ciliary tip kinesins prior to their entry into cilia. We propose that loss of Arl3 leads to a failure of ciliary tip kinesin delivery to the cilia base, while loss of RP2 leads to a failure of efficient release of kinesins from Arl3. A recent study showed that knockdown of Arl13b resulted in the accumulation of IFT proteins at the ciliary tip, possibly due to impaired retrograde cilia protein trafficking (58). In addition a role for Arl13b in stabilising the association of the IFT-A and IFT-B complex has been proposed in *C. elegans*, as loss of Arl13 leads to dissociation of the IFT-A and IFT-B complex (24); however, in the absence of Arl13b these phenotypes could also be caused through the accumulation of GDP-Arl3 in the ciliary compartment.

The role of Kif7 and Kif17 in photoreceptors is currently unknown. Deletion of Kif17 in mouse or zebrafish does not appear to affect photoreceptor viability or protein traffic (17,20), and therefore the reason Kif17 accumulates at the tip of the photoreceptor is unknown. Mutations in *Kif7* cause Joubert syndrome and acrocallosal syndrome in man (59,60), and are embryonic lethal in mouse (61). The retina is not commonly affected by mutations in *Kif7*; however, it has been suggested to be a potential modifier of other ciliopathies (60). Our data show that Kif7 is localized at the ciliary tip in developing photoreceptors and could play a role in stabilizing the outer segment axoneme or facilitating outer segment function. Therefore the role of Kif7 in photoreceptors warrants further investigation.

We have shown previously that PTC124 and G418 are able to restore functional, full-length RP2 in fibroblasts and RPE cells (41). In this study, we were able to rescue the targeting of Gli3 and Kif7 at cilia tips in RP2 R120X fibroblasts and Kif7 in iPSC derived photoreceptors using TRIDs. G418 was toxic to optic cups, highlighting the known hazards of aminoglycosides. In contrast, PTC124 (also known as ataluren) is well tolerated and has EMA and FDA orphan drug designation for the treatment of Duchenne Muscular Dystrophy and Cystic Fibrosis caused by nonsense mutations. Our data provide further evidence that PTC124 might be a safe and effective approach to treat nonsense mutations in RP2 and restore RP2 protein function.

## Materials and Methods

### Plasmids and transfections

Human telomerase reverse transcriptase retinal pigment epithelial (hTERT-RPE) cells and Chinese hamster ovary (CHO) cells were grown in Dulbecco’s modified Eagle’s medium (DMEM)/F12 (Invitrogen, Paisley, UK). Serum rich medium contained additionally 10% (v/v) foetal bovine serum (FBS). Cell cultures were not treated with antibiotics. For maintenance, cells were passaged every 3-4 days or when they were approximately 90% confluent. For immunofluorescence staining, cells were cultured in 8- well chamber slides (VWR, Lutterworth, UK). Cells for Western blotting were plated into 6-well plates (Nunc, Fisher Scientific, UK). Transfections of plasmids were performed using Lipofectamine and Lipofectamine Plus reagent according to the manufacturer’s instructions (Invitrogen).

Human GFP-Kif17 and myc-Kif17 constructs were a kind gift from Prof Geri E. Kreitzer (Weill Cornell Medical College, New York, USA). The human full-length V5-Kif7 construct was a kind gift from Dr Max Liebau, University of Cologne, Germany. The full-length, human GFP-Kif3A plasmid was purchased from Addgene. Generation of RP2-GFP and Arl3 GDP and GTP locked confirmations has been described elsewhere (31,62). To create hTERT-RPE cells stably expressing human GFP-Kif17 plasmids, cells were transfected with 1 μg of plasmid using Lipofectamine and Lipofectamine Plus reagents. Cells were subsequently cultured in selection medium (DMEM/F12, 10% FBS, 0.6 mg/ml G418) and low expressing green fluorescent cell colonies were isolated manually and further expanded.

### RNA interference

For RNA interference studies hTERT-RPE cells were plated either plated into 8-well chamber slides (VWR) for ICC or 6-well plates (Nunc) for analysis of protein knockdown. A pool of four small interfering RNAs (siRNA) for RP2, Arl3, Kif17, Kif7 and a non-targeting control siRNA were obtained from Dharmacon (ON-TARGET plus siRNA reagents, Chicago, USA). The sequences of the control, RP2 and Arl3 siRNAs (sense strands) have been published previously (31). The sequences for the Kif17 siRNA were as follows: *5’ GCAACUACUUCCGAUCUAA, 5’ CGAGAUGUCUGCCGUGGAU, 5’ CCACAUCCCGUCAUCACAA, 5’ CGACAUCCCUUUCACCAAG*. The sequences for the Kif7 siRNA were: *5’ GGAUGAUUGAUGUCCGGAA, 5’ UGCAGGAGCUCGAGCGGAA, 5’ GCCUGGAGAUCGACGGCAA, 5’ GCAGAUUGCCUUCUCGGAA.*

### Antibodies and western blotting

RP2 protein was detected using the crude sheep anti-RP2 antibody (1:2000) (62). The rabbit anti-Arl3 antibody (1:2000) was a kind gift from Dr. N. J. Cowan (New York University, USA). The rabbit anti-Kif17 antibody (1:3000) was purchased from Sigma-Aldrich. GAPDH protein expression was detected using the mouse anti-GAPDH antibody (1:10000, Sigma-Aldrich). Mouse anti-pericentrin (1:2000, Abcam), Arl13b (1:1000, ProteinTech), acetylated alpha tubulin (1:1000, Sigma Aldrich) and polyglutamylated tubulin (1:1000, Adipogen) were used as basal body and ciliary markers, respectively. The Kif7 antibody used for ICC was a kind gift from Prof Kathryn Anderson (Sloane Kettering Institute, New York, USA), while the Kif7 antibody used for Western blotting was purchased from the Protein Tech Group (1:1000). The Gli3 antibody (1:1000) was obtained from R&D Systems. The Kif3B antibody (1:1000) was purchased from Santa Cruz. 3D optic cups were stained with recoverin (1:500, Millipore), Sox2 (1:1000, Abcam), Nestin (1:200, Abcam), Chx10 (1:300, Santa Cruz), HuD (1:300, Santa Cruz) and cone arrestin 7G6 (1:500, gift from Dr. Cheryl Craft, USC, USA). Secondary antibodies used were horseradish peroxidase (HRP) conjugated goat anti-sheep, donkey anti-rabbit or goat anti-mouse antibodies (Stratech). Blots were developed using the enhanced chemiluminescence (ECL) Western blotting detection system (GE Healthcare). Protein levels were quantified using the ImageJ software.

### Co-immunopurification

Co-immunopurification (co-IPs) were performed using magnetic Dynabeads Protein G (Invitrogen) or GFP-Trap_M beads (Chromotek, Germany) according to manufacturer’s instructions. Briefly, CHO cells were plated into 6-well plates and transfected as described above. Cells were lysed in lysis buffer (25 mM Tris pH 7.5, 150 mM NaCl, 0.25% deoxycholate, 1% NP-40, 1 mM EDTA, PIC, PhlC) and lysates were subsequently incubated with 25 μl of Dynabeads and primary antibodies or 25 μl of GFP-coupled magnetic beads (Chromotek, Germany) for 1 h at room temperature on a rotating wheel. The beads were washed several times with lysis buffer before elution with 2x SDS loading buffer and analysis by Western blotting.

### Immunocytochemistry 

For immunocytochemistry (ICC), cells were washed twice in PBS and either fixed in 100% ice-cold methanol for 2 min or 4% paraformaldehyde (PFA) for 10 min. Cells were permeabilized with 0.2% Triton X-100 in PBS for 10 min and subsequently incubated for 1 h in blocking buffer (3% bovine serum albumin (BSA), 10% normal donkey serum in PBS) to avoid non-specific antibody binding. Following block, cells were incubated for 1 h with primary antibodies at the appropriate titre. After washing with PBS cells were then incubated with fluorescent labelled (Cyanine2 or Cyanine3) secondary antibodies (1:100, Stratech, Newmarket, Suffolk, UK) for 1 h in 3% BSA in PBS. Following several washes with PBS cells were incubated for 5 min with 2 μg/ml 4',6-diamidino-2- phenylindole (DAPI, Sigma-Aldrich) in PBS to stain the nuclei. Confocal images were obtained using the LSM700 microscope (Carl Zeiss MicroImaging) and analysed using the LSM Image Browser software (Carl Zeiss MicroImaging), prior to export and image processing and annotation using Adobe Photoshop and Illustrator. Fluorescence of GFP-Kif17 at the ciliary tip was analysed in ImageJ and correct total cell fluorescence calculated using an established protocol (63). Line scan analysis of cilia was performed using ImageJ. A Zeiss ELYRA microscope was used to perform super-resolution structured illumination (SIM). Sample preparation for SIM was identical to that for standard confocal except that cells were cultured on high-precision cover glasses (Zeiss).

### iPSC and optic cups

Reprogramming of R120X patient and control iPSC have been described elsewhere (41). iPSC were differentiated into photoreceptors and optic cups using methods based on those described previously with some modifications (48). Briefly embryoid bodies were generated from iPSC and transitioned to neural induction media (DMEM-F12, 1% N2, 1% NEAA). Embryoid bodies were seeded onto geltrex (Thermofisher) coated 6 well plates and transitioned to retinal differentiation medium (DMEM-F12, 2% B27, 1% NEAA). After 12 weeks transparent neuroepithelial domains and RPE were isolated with a pipette tip and cultured in suspension in neural retina media (DMEM-F12, 1% N2, 10% FBS, 0.5μm retinoic acid) for a further 8 weeks. After 21 weeks total differentiation time optic cups were fixed in 4% PFA, mounted in OCT and cryosectioned into 6μm slices.

### Statistical analysis

To measure fluorescence intensity, images were analysed in ImageJ. Statistical analyses were performed using the excel software and significance determined using the Student’s t test. Data are presented as means ± SEM. Significance levels were set when *P < *0.05 (*), *P < *0.01 (**), *P < *0.001 (***).

## Supplementary Material

Supplementary Data
