# Arl3 and RP2 regulate the trafficking of ciliary tip kinesins

**DOI:** 10.1093/hmg/ddx245

**Published:** 2017-07-10

**Authors:** Nele Schwarz, Amelia Lane, Katarina Jovanovic, David A. Parfitt, Monica Aguila, Clare L. Thompson, Lyndon da Cruz, Peter J. Coffey, J. Paul Chapple, Alison J. Hardcastle, Michael E. Cheetham

**Affiliations:** 1 UCL Institute of Ophthalmology, London EC1V 9EL, UK; 2 Institute of Bioengineering, School of Engineering and Materials Science, Queen Mary University of London, London E1 4NS, UK,; 3 Moorfields Eye Hospital, London EC1V 2PD, UK; 4 William Harvey Research Institute, Barts and the London School of Medicine, Queen Mary University of London, London EC1M 6BQ, UK

*Human Molecular Genetics* 2017, 26, 2480–2492.

doi: 10.1093/hmg/ddx143

The Authors would like to add NC3Rs to the funders acknowledged in their paper.

This has also been corrected online.

