# Supplementary material for: Arl3 and RP2 regulate the trafficking of ciliary tip kinesins
Source: Hum Mol Genet. 2017 Apr 21;26(13):2480–92. doi: 10.1093/hmg/ddx143 (PMC5808637; doi:10.1093/hmg/ddx143)
Supplement: Supplementary Data [file ddx143_supp.pdf]

Arl3 and RP2 regulate the trafficking of ciliary tip kinesins

Schwarz et al

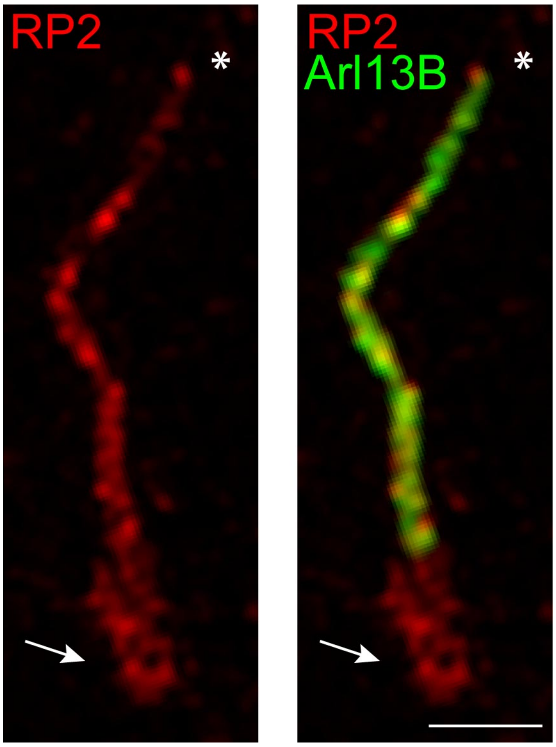

Supplementary material Figure 1. RP2 localises to the basal body and along the length of cilia. Structured Illumination Microscopy (SIM) revealed that endogenous RP2 (red) colocalised with the ciliary marker Arl13b (green) in cilia as well as accumulating at the basal body. Arrows indicate basal body; asterisks indicate cilia tip. Scale bar 0.5 µm.

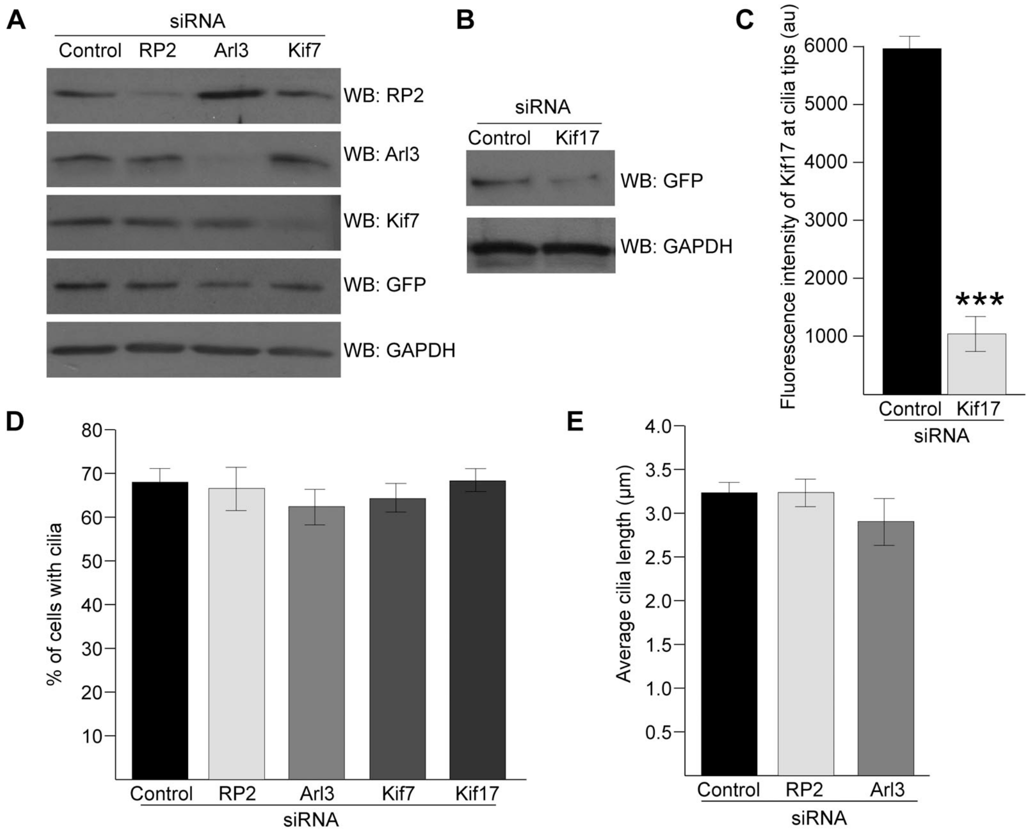

Supplementary material Figure 2. Efficiency of knockdown and phenotypic effect of siRNAs. (A) Western blot of GFP-Kif17 hTERT-RPE stable cells transfected with different siRNAs showing efficient target knockdown without affecting GFP-Kif17 levels. **(B)** Western blot of GFP-Kif17 cells treated with control or Kif17 siRNA shows knockdown of Kif17 protein levels. **(C)** Quantification of GFP-Kif17 fluorescence intensity at cilia tips following treatment with control of Kif17 siRNA. n≥150 cilia analysed from 3 independent experiments. ***P≤0.001; values are mean ± SEM. Quantification of cilia incidence **(D)** and length **(E)** in hTERT-RPE cells with different siRNAs. n≥150 cilia analysed from 3 independent experiments. Values are mean ± SEM.

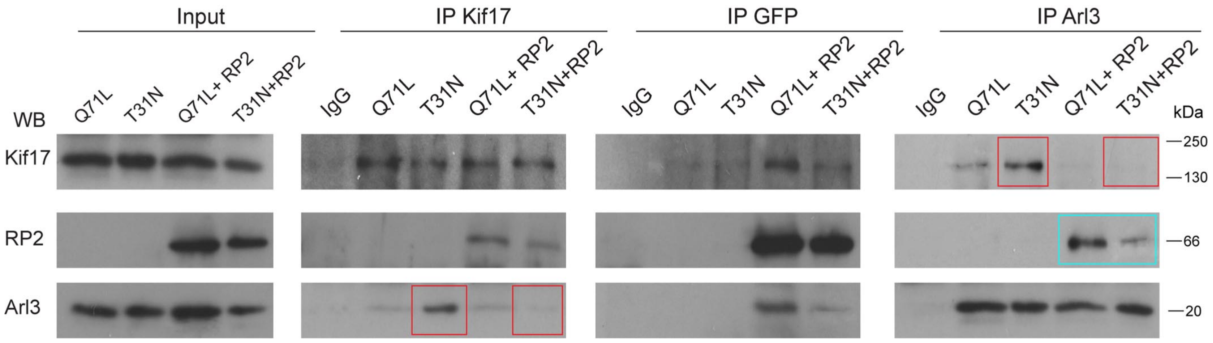

Supplementary material Figure 3. RP2 competes with the GDP-Arl3 for Kif17 binding. RP2 competes successfully with the GDP conformational mimic of Arl3 (T31N) for binding to GFP-Kif17, as highlighted by the red boxes. RP2 binds preferentially to GTP-bound Arl3 (Q71L) as highlighted by the blue box.

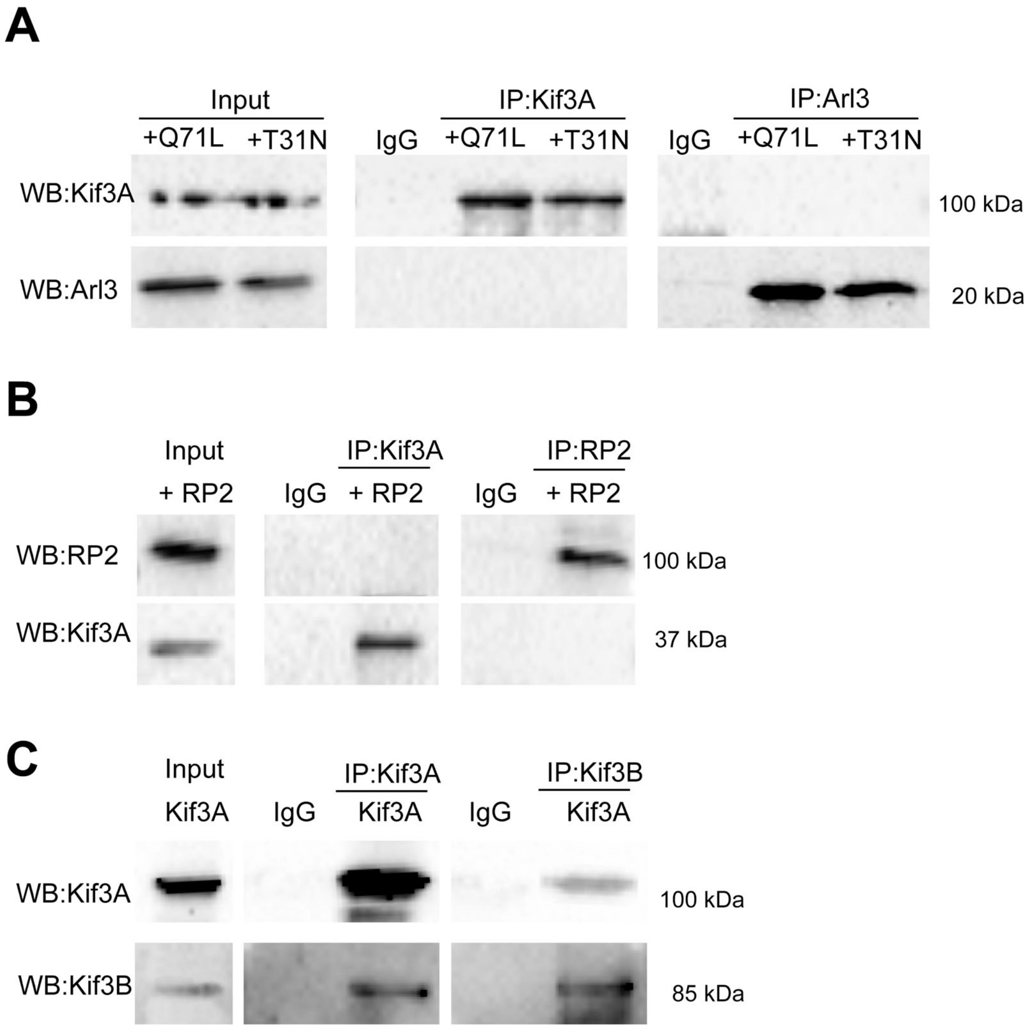

Supplementary material Figure 4. RP2 and Arl3 do not interact with Kif3A. Reciprocal co-IP showing that GFP-Kif3A does not bind to the GDP or GTP conformational mimic of myc-Arl3 (T31N and Q71L, respectively) **(A),** or to RP2 **(B)**. Kif3A and Arl3 were immunopurified with antibodies against GFP and myc, respectively. **(C)** Reciprocal co-IP showing that GFP-Kif3A binds to Kif3B. Kif3A was immunopurified with the GFP antibody.

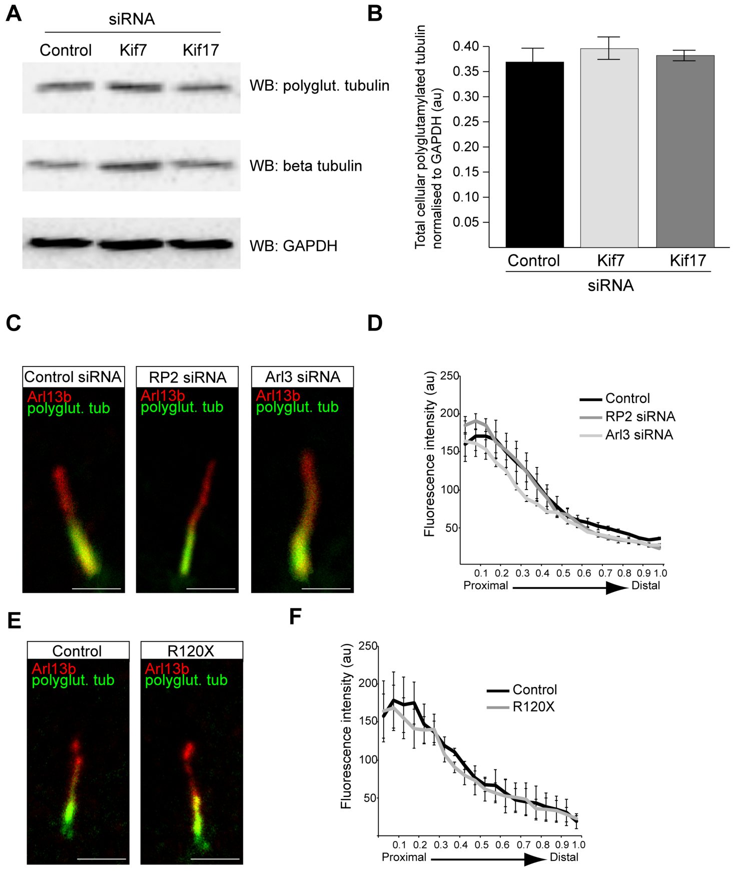

Supplementary material Figure 5. Effect of siRNA treatment on cellular and cilia polyglutamylation. (A) Western blot showing total cellular polyglutamylation levels are unchanged following siRNA treatment with Kif7, Kif17 or control siRNA. **(B)** Quantification of Western blot. Total polyglutamylation was normalised to GAPDH. n=2 independent experiments. **(C)** Loss of RP2 and Arl3 do not affect cilia stability. Transfection of hTERT-RPE cells with siRNA against RP2 or Arl3 did not affect levels of polyglutamylated tubulin (green). Cilia marker Arl13b (red). Scale bar 1 μm. **(D** Fluorescent intensity of polyglutamylated tubulin, as shown in (C) normalised for cilium length. n=3 independent experiments, with a total of 90 cilia measured for each siRNA treatment. P=n.s., values are mean ± SEM. **(E)** Control and RP2 R120X patient fibroblasts stained for polyglutamylated tubulin (green) and Arl13b (red). Scale bar 1 μm. **(F)** Fluorescent intensity of polyglutamylated tubulin, as shown in (**E**) normalised for cilium length. n=3 independent experiments, with a total of 90 cilia measured for each siRNA treatment. P=n.s., values are mean ± SEM.

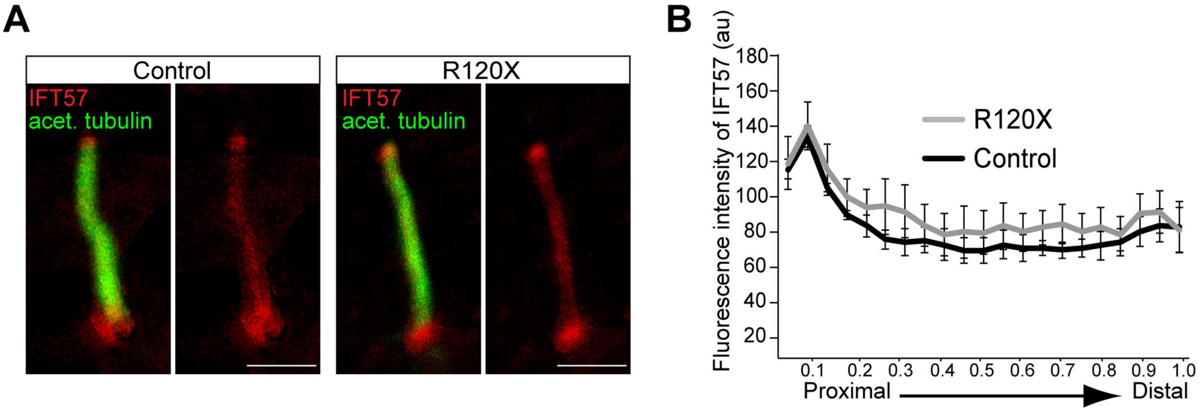

Supplementary material Figure 6. IFT-B2 subunit localisation is not affected by Arl3 or RP2 siRNA. (A) Control and RP2 R120X fibroblasts were stained for Kif7 (red) and the cilia marker acetylated α-tubulin (green). Scale bar 1 μm. **(B)** Fluorescence intensity of IFT57, as shown in (A) normalised for cilium length. n=3 independent experiments, with a total of 90 cilia measured for each fibroblast cell line. P=n.s., values are mean ± SEM.

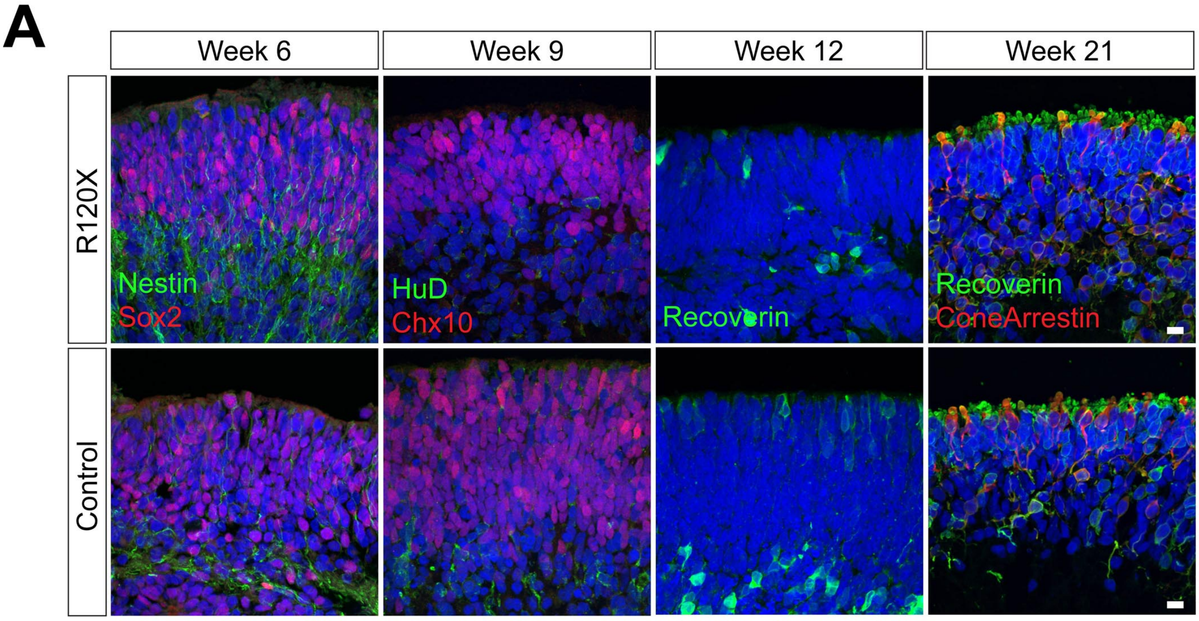

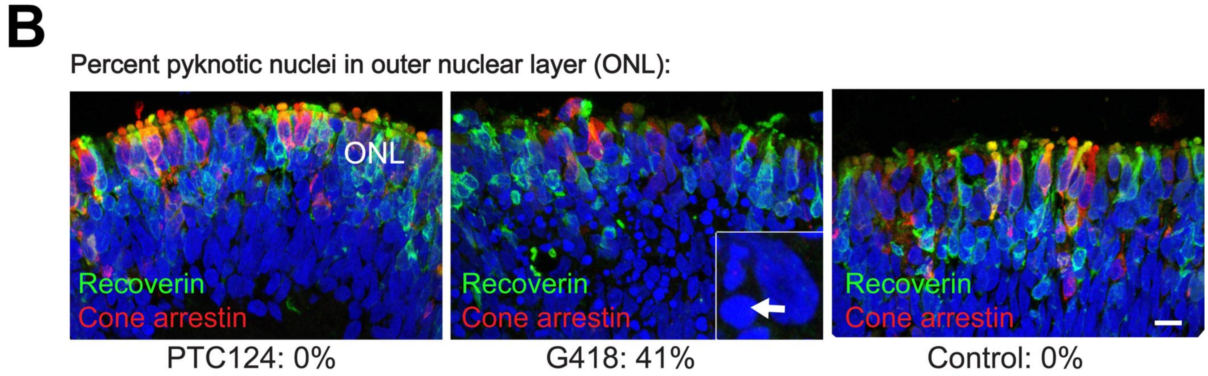

Supplementary material Figure 7. Phenotypic analysis of iPSC-derived optic cups (A) R120X optic cups displayed no discernible developmental defects with timely expression of key markers or retinal development compared to controls. Markers of neuroepitelial progenitors, Nestin (green) and Sox2 (red) are present at week 6, followed by markers of neuro-retinal progenitors and ganglion cells, Chx10 (red) and HuD (green) at week 9, and rod and cone photoreceptors labelled with recoverin (green) and cone arrestin (red) which increase in abundance from weeks 12 -21. Scale bar = 10 µm. **(B)** G418 is toxic to 3D optic cups. Day 150 RP2 R120X optic cups were treated with three doses of 10 µg/ml PTC124, 500 µM G418 or vehicle control. Photoreceptors in the outer nuclear layer were stained with recoverin (green) and cone arrestin (red). Inset in G418 shows magnification of pyknotic nuclei in the outer nuclear layer (ONL). Scale bar 10 µm.

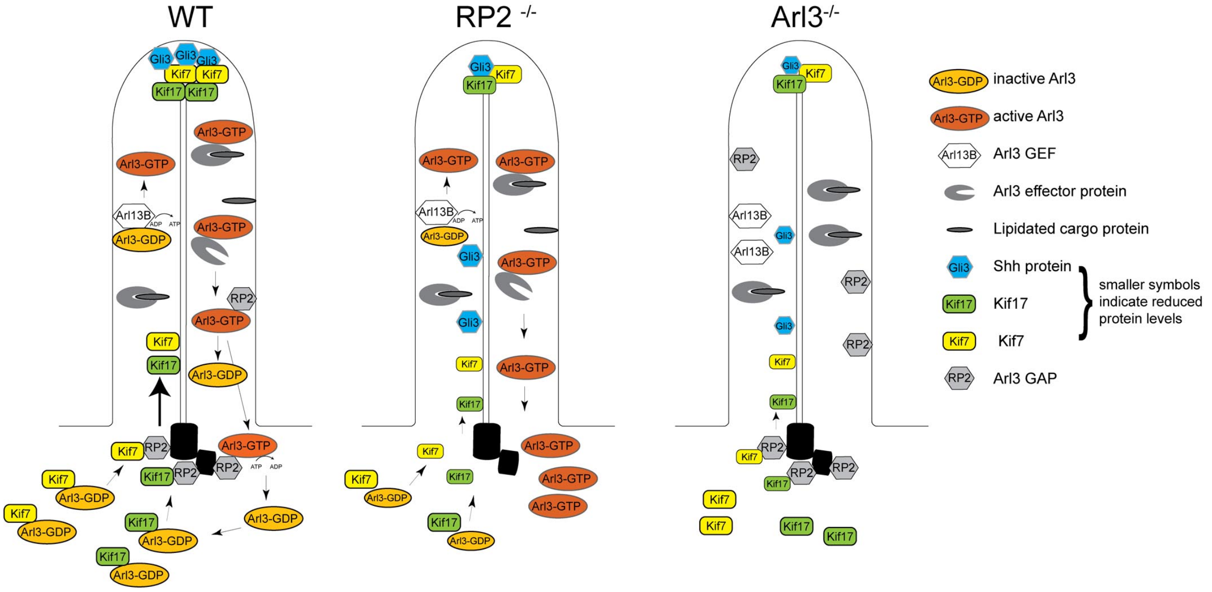

Supplementary material Figure 8. Model of Arl3 and RP2 mediated trafficking of ciliary tip kinesins. RP2 competes with Arl3-GDP for binding of Kif17, and potentially Kif7, at the basal body of cilia. Arl3-GDP is then released from Kif7 or Kif17 binding and enters cilia, where its GEF Arl13b converts it from Arl3-GDP to Arl3-GTP. Arl3-GTP is then able to release cargo proteins from effector proteins, as proposed previously (25). Kif7 shapes the ciliary tip compartment to allow the accumulation of Gli3 and Kif17. Upon exit from cilia Arl3-GTP is converted to Arl3-GDP at the ciliary base and can bind Kif7 or Kif17 in the next trafficking cycle. Loss of RP2 results in insufficient release of Kif7 and Kif17 from Arl3, and therefore reduced ciliary entry of these proteins. Loss of Arl3 prevents efficient delivery of ciliary tip kinesins to the basal body, which in turn causes the reduction of these proteins at cilia tips and reduced Gli3 levels.
